# Experimental Investigation of a Wing-in-Ground Effect Craft

**DOI:** 10.1155/2014/489308

**Published:** 2014-02-16

**Authors:** M. Mobassher Tofa, Adi Maimun, Yasser M. Ahmed, Saeed Jamei, Agoes Priyanto, Rahimuddin

**Affiliations:** ^1^Faculty of Mechanical Engineering, Universiti Teknologi Malaysia, 81310 Skudai, Johor, Malaysia; ^2^Marine Technology Centre, Universiti Teknologi Malaysia, 81310 Skudai, Johor, Malaysia

## Abstract

The aerodynamic characteristics of the wing-in-ground effect (WIG) craft model that has a noble configuration of a compound wing was experimentally investigated and Universiti Teknologi Malaysia (UTM) wind tunnel with and without endplates. Lift and drag forces, pitching moment coefficients, and the centre of pressure were measured with respect to the ground clearance and the wing angle of attack. The ground effect and the existence of the endplates increase the wing lift-to-drag ratio at low ground clearance. The results of this research work show new proposed design of the WIG craft with compound wing and endplates, which can clearly increase the aerodynamic efficiency without compromising the longitudinal stability. The use of WIG craft is representing an ambitious technology that will help in reducing time, effort, and money of the conventional marine transportation in the future.

## 1. Introduction 

Wing in ground effect is quite a new concept of designing fast ships, which has vast relevance in numerous areas such as transportation of cargo, tourism, rescue operations, and military functions. WIG craft gives an alternate solution to gain higher speed [1]. The ground effect (GE) is a phenomenon where the lift-to-drag ratio of a body will increase while it is cruising at a very close distance to the surface of water or ground [2]. Volkov and Russetsky [3] and Hooker [4] widely discussed useful characteristics of WIG craft. The development of WIG crafts originated from observations made of the landing performance of aircraft in 1920's. Later USA and the USSR became interested in attempting to exploit the potential benefits of ground effect. The USA abandoned efforts to produce ground effect craft in the mid 1960's as they were more interested in surface effect ship development. Germany began work in the late 1960's using the designs of Alexander Lippisch. However, USSR was the undisputed leader in research and development of WIG crafts up to the late 1980's [5]. Under these circumstances, the Ministry of Science Technology and Innovation (MOSTI) Malaysia provided funds to develop a WIG craft here.

**Figure 1:**
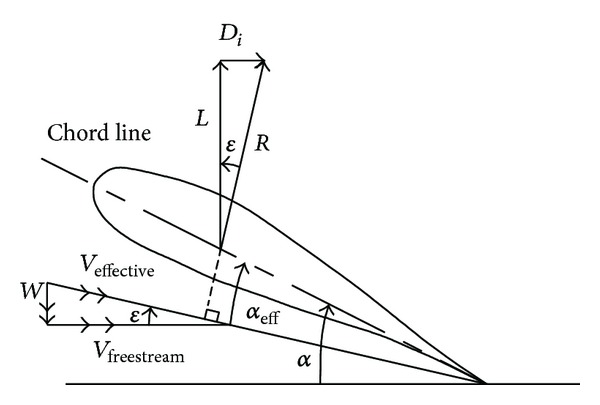
Lift and drag of a wing section [6].

The lift and drag that are produced by a wing define the performance and general attributes of the craft that it supports. A wing that is moving through the air produces a resultant force. Lift is the component of the resultant force perpendicular to the velocity vector of the wing. The component that is resultant force parallel to the velocity vector of the wing is defined as induced drag. There are other forms of drag, which are collectively known as parasite or profile drag; this drag is created by the friction of the object moving through the air. The total drag of an object moving through the air, can be achieved by summing up induced drag and parasite drag. Both lift and drag are functions of a number of variables such as the density of the air, the velocity of the object through the air and the geometry of the object.  Figure 1 depicts the formation of lift (*L*) and induced drag (*D*_*i*_) from the resultant force (*R*) created by the wing's movement through the air. In addition, the previous figure shows that the position of the wing as it moves through the air is characterized by the geometric angle of incidence (*α*), where the geometric angle of incidence is the angle between the chord line of the wing section and the velocity vector of the wing [6]. If the wing is designed properly, the lifting surface near ground generates higher lift at smaller ground clearances. However, the drag decreases with decreasing ground clearance for span-dominated GE.

A properly designed lifting system should increase lift-to-drag ratio and reduce drag for constant lift as the ground clearance reduces [5]. Many experimental and numerical simulations have been conducted by several researchers to study the phenomenon of GE. Jung et al. [7] conducted experimental investigation of wing with NACA 6409 section. Three different types of endplates were tested in those experiments at various ground clearances, angle of attacks, and aspect ratios. Endplates help to increase the performance of the wing though moving its center of pressure forward to the leading edge. Furthermore, it was found that the freestream velocity has insignificant effect on the wing aerodynamic coefficients. Pressure distribution through the symmetrical airfoil surface under different ground clearances and angle of attack were studied by Ahmed and Sharma [8]. NACA4412 airfoil section with flap in extreme ground effect were numerically investigated by Ockfen and Matveev [9]. The study consists of a steady-state, incompressible, finite volume method using Spalart-Allmaras turbulence model for the turbulent flow. It was found that favorable trailing-edge flap configuration improves aerodynamic characteristics of NACA4412 wing section. Chun and Chang [10] numerically analyzed the turbulent flow around two-dimensional WIG with respect to two ground boundary conditions, that is, moving and fixed bottom boundary. According to this study, the lift force and moment are not influenced by different bottom conditions, though the drag force simulated by the moving bottom is greater than that by the fixed one. The influence of the endplates on the aerodynamic characteristics of small aspect ratio (AR) wing was studied by Park and Lee [11]. According to their study, the reduction of the tip vortex will develop a substantial rise in the lift and lift-to-drag ratio, which can be developed by endplate. They claimed endplates also made a small deviation of height stability at different ground clearances and angle of attacks, which can reduce the height stability. Park and Lee [12] demonstrated the optimal profiles of two-dimensional wings to reach a moderate stability and high efficiency. In an another research work by Yang et al. [13], it was shown that for three-dimensional wing, end plates actually enhance the height stability at low ground clearance. Kornev and Matveev [14] found that the profiles of tail wing and main wing are the main factors of static height stability. They suggested that for acceptable stability of a WIG craft, the centre of gravity should be close to the height of aerodynamic center (*X*_*h*_), and it should be between the height of aerodynamic center and pitch aerodynamic center (*X*_*α*_). Irodov [15] recommended a height static stability criterion as follows:

(1) HS=CMαCLα−CMzCLz<0,Xα=CMαCLα,Xh=CMzCLz,

where *CM*_*α*_, *CL*_*α*_, *CM*_*z*_, and *CL*_*z*_ are derivatives of lift and moment coefficient with respect to pitching angle and height. In a stable WIG craft, these derivatives usually are *CL*_*z*_ > 0, *CM*_*α*_ < 0, *CL*_*α*_ > 0, and *CM*_*z*_ > 0 [15].

A new configuration of the compound wing was developed in UTM by Jamei et al. [16] to improve the aerodynamic performance of the wing. The compound wing was divided into three parts: the middle part of rectangular wing shape and the side parts with reverse taper wings with an anhedral angle. The compound wings could create a greater reduction of downwash velocity and modify the pressure distribution on the lower side. The high increment of lift-to-drag ratio for the present wing in extreme ground effect recognizes a good efficiency for wing-in-ground (WIG) craft. Furthermore, it has been found that the performance of the wing improves noticeably for a certain total span of compound wing when the span of the middle part becomes smaller. It can be seen from the literature review that improving the aerodynamic characteristics of the wing in proximity to the ground by different means, such as the endplates, different configuration of wing is one of the important parameters in designing a WIG craft. The improvement of wing can increase the range and endurance of flight and decrease the fuel consumption and CO_2_ emission. Therefore, there are many research articles dealt with the wing in ground effect, but most of these articles focused on the aerodynamic characteristics of the wing only and not the whole WIG craft.

In this research work, the aerodynamic characteristics and static stability of the WIG craft with the compound wing configuration [14] developed at UTM was experimentally investigated with and without endplates.

## 2. UTM Wind Tunnel

The aerodynamic characteristics, specifically the ground effect, of the WIG craft with a compound wing were investigated in a low speed wind tunnel at the Universiti Teknologi Malaysia (UTM-LST). This wind tunnel was able to deliver a maximum air speed of 80 m/s (160 knots or 288 km/hr) inside the test section. The size of test section was 2.0 meters wide, 1.5 meters height, and 5.5 meters long. The flow inside the wind tunnel was of good quality, with a flow uniformity <0.15%, temperature uniformity <0.2, flow angularity uniformity <0.15, and turbulence <0.06%. UTM-LST had high-quality facilities that allow for accuracy and repeatability of experiment results [17].

The wind tunnel is equipped with a 6-component balance for load measurements. The balance is a pyramidal type with virtual balance moment at the centre of the test section. The balance has a capability to measure aerodynamic forces and moment in the 3-dimensional. The aerodynamic loads can be tested at various wind direction by rotating the model via turntable. The accuracy of the balance is within 0.04% based on 1 standard deviation. The maximum load range is ±1200 N for axial and side loads [17].

## 3. WIG Craft Model

**Figure 2:**
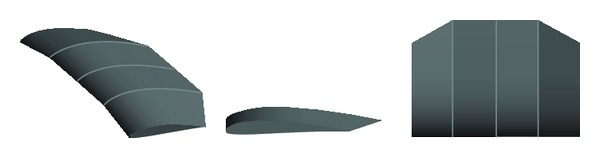
New compound wing configuration designed in UTM [16].

**Figure 3:**
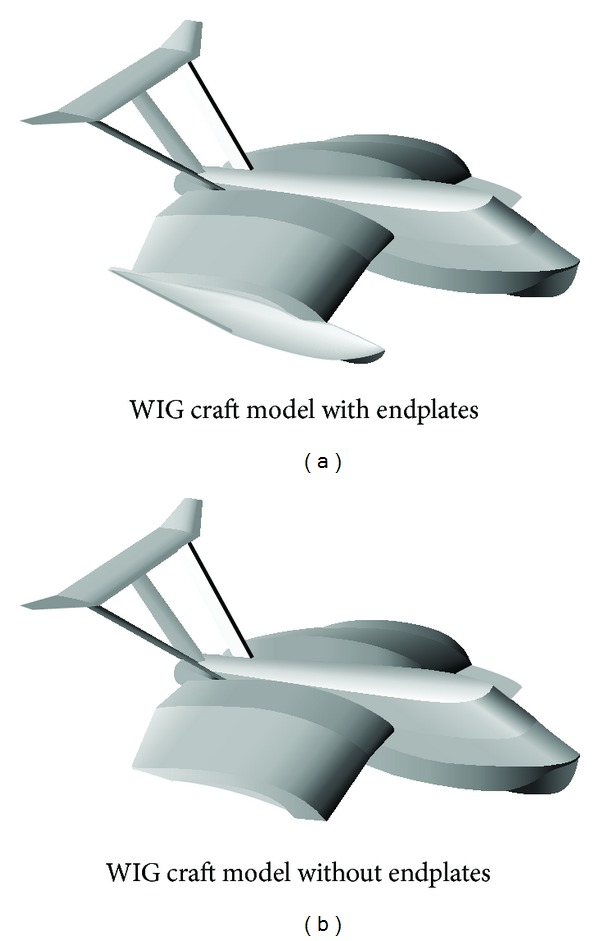
WIG craft model with compound wing.

The experiments were carried out on a WIG craft model that used a new compound wing configuration [16] as shown in Figures 2 and 3. The compound wing was composed of three parts: a rectangular wing in the middle and two reverse taper wings with an anhedral angle at the sides [16]. The NACA 6409 airfoil section was selected as a section of the compound wings. The principal dimensions of the WIG craft are summarized in Table 1.

## 4. Experimental Procedures and Set-Up

In the wind tunnel, aerodynamic force measurements were carried out for a range of ground clearances (*h*/*c*) and different angles of attacks (*α*), from *h*/*c* = 0.18 to *h*/*c* = 0.25 and from *α* = 4° to *α* = 6°. Ground clearance (*h*/*c*) was defined as the distance ratio between the wing trailing edge centre and ground surface (*h*) to root chord length (*c*) of the wing.

**Figure 4:**
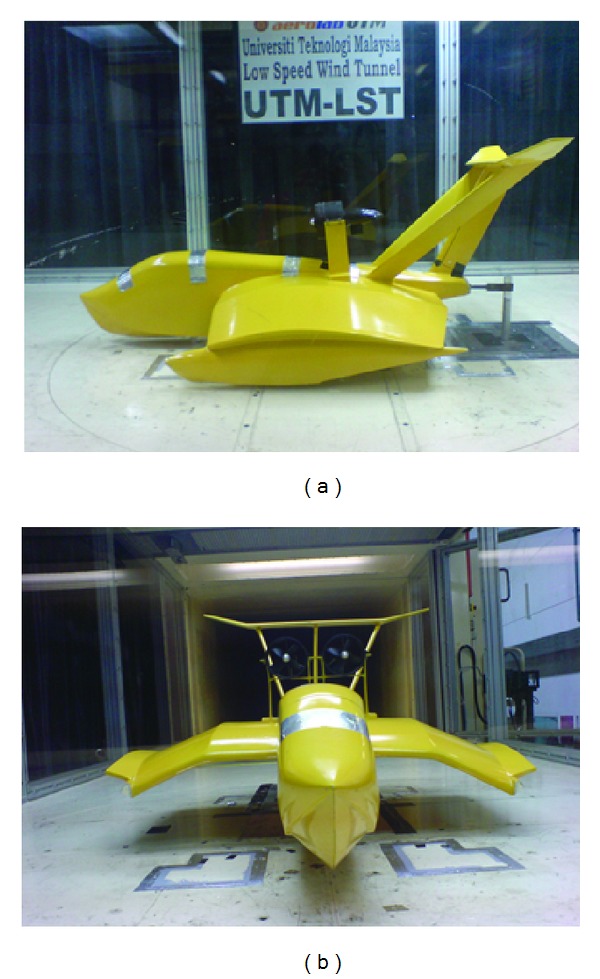
Wind tunnel test for WIG craft model: (a) model with endplates and (b) model without endplates.

**Figure 5:**
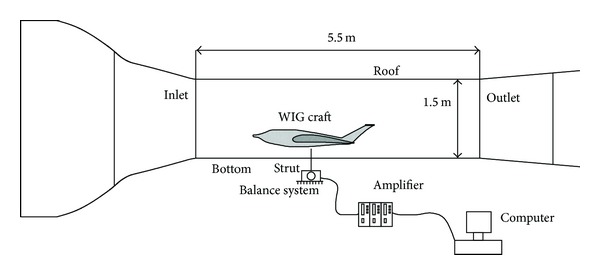
Experimental setup in the wind tunnel at the Universiti Teknologi Malaysia.

In this study, the floor of wind tunnel was used as a fixed flat ground as shown in Figure 4. The WIG craft model was mounted on the test section of the wind tunnel with a strut as shown in Figure 5. The position of the strut was at the 40% of chord length from the leading edge of the compound wing. The strut was adjustable and then was fixed at any height. The WIG craft model could be rotated about an axis at the strut position.

The frontal area ratio of the WIG craft model and the test section was small; therefore, the blockage ratio for the wings related to side and roof walls of the wind tunnel can be neglected. In this study, all experiments were performed with freestream velocity of 25 m/s.

## 5. Results and Comparisons 

The WIG craft model aerodynamic coefficients and centre of pressure were obtained in this study using the following formulas:

(2) CL=L0.5ρU2S,CD=D0.5ρU2S,CM=M0.5ρU2Sc,Xcp=0.4+CMCLcos⁡ α+CDsin α.

**Figure 6:**
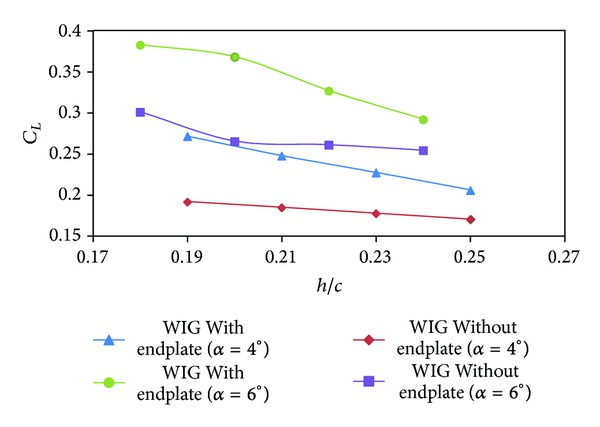
Lift coefficient (*C*_*L*_) of WIG craft model with and without endplates versus ground clearance (*h*/*c*) at different angles of attack.

The lift coefficients of the WIG craft model with and without endplates are shown in Figure 6. As expected the lift coefficient of the WIG craft model with endplates was higher than the lift coefficient of the WIG craft model without endplates. The lift coefficients of the WIG craft model increase up to 42% and 50% for angles of attack of 4° and 6°, respectively. From the previous figure, it is clear for higher angle of attack and lower ground clearance endplates significantly enhanced the lift coefficient.

**Figure 7:**
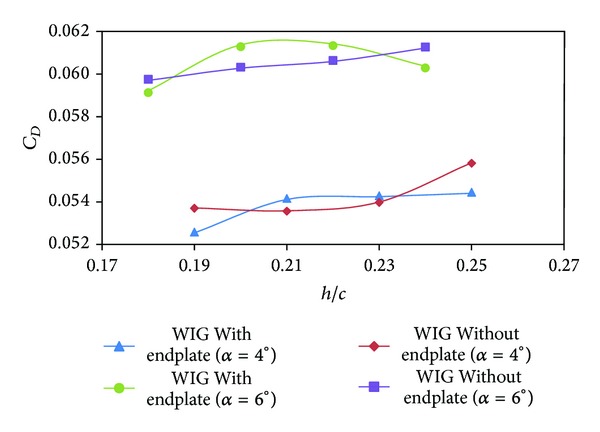
Drag coefficient (*C*_*D*_) of WIG craft model with and without endplates versus ground clearance (*h*/*c*) at different angles of attack.

Figure 7 depicts the drag coefficient (*C*_*D*_) of the WIG craft model with and without endplates. The drag coefficients of the WIG craft model with and without endplates do not show high discrepancy for each angle of attack, probably because of reduction of induced drag due to the effect of endplates. Thus, the increment of drag can be related only to angle of attack and ground clearance. For higher angle of attack and ground clearance drag coefficient increases up to 11% and 5%, respectively, as shown in the figure.

**Figure 8:**
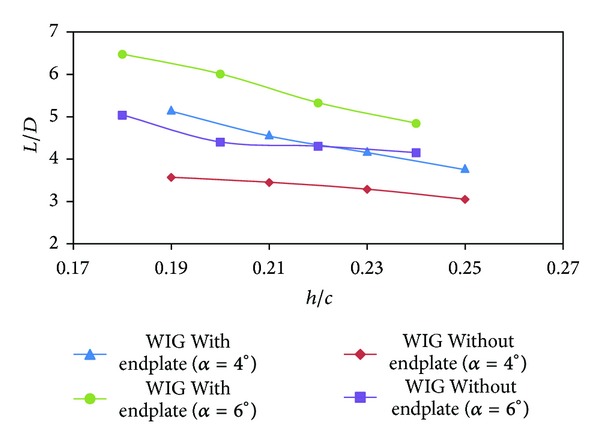
Lift-to-drag ratio (*L*/*D*) of WIG craft model with and without endplates versus ground clearance (*h*/*c*) at different angles of attack.

The performance of an aircraft is defined by its lift to drag ratio (*L*/*D*). The comparison of lift to drag ratio between the WIG craft model with and without endplates is shown in Figure 8. For lower ground clearance endplates enhance the lift-to-drag ratio up to 45%; the lift-to-drag ratio of WIG craft model with endplates decreases sharply compared to the model without endplates as the ground clearance increases. Augmentation of lift to drag ratio can also be attributed to the angle of attack, where larger angle of attack amplifies the lift to drag ratio. In addition, *L*/*D* decreased when the ground clearance was smaller.

**Figure 9:**
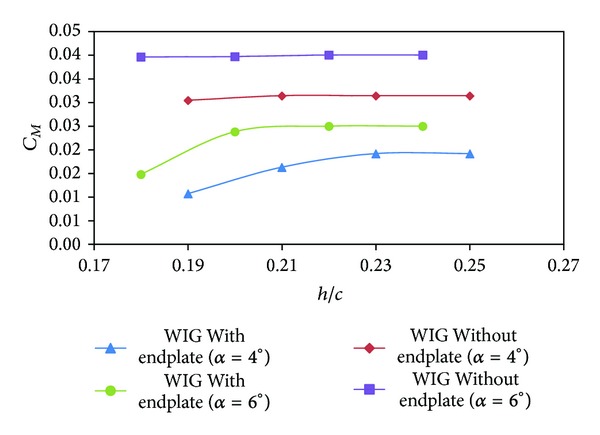
Moment coefficient (*C*_*M*_) of WIG craft model with and without endplates versus ground clearance (*h*/*c*) at different angles of attack.

The moment coefficients (*C*_*M*_) of the WIG craft model with and without endplates are shown in Figure 9. The pitching moments were measured about the point which was 40% of the chord length from the leading edge. The *CM*_*z*_ being positive for the wing with end plates is consistent with the prior discussion about stability for a WIG.

**Figure 10:**
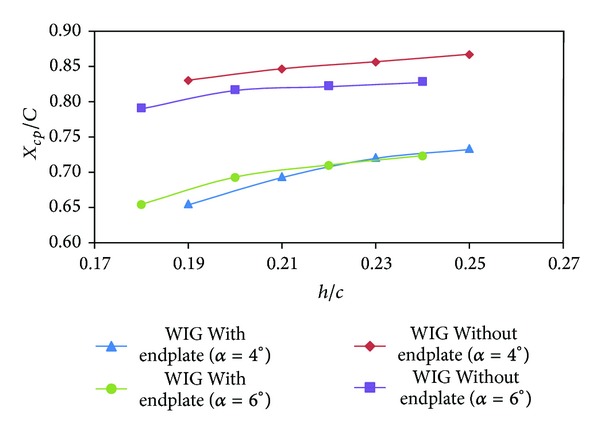
The position of the center of pressure (*X*_*cp*_) of WIG craft model with and without endplates versus ground clearance (*h*/*c*) at different angles of attack.

The second parameter that has main effect on the stability of  WIG craft is its center of pressure. The distance between the leading edge and center of pressure on the wing is defined as *X*_*cp*_.  Figure 10 shows the center of pressure for the WIG craft model with and without endplates. In general, the position of center of pressure of the WIG craft model shifted towards leading edge due to effect of endplates. This effect is expected as pitching moment reduced due to endplates.

**Figure 11:**
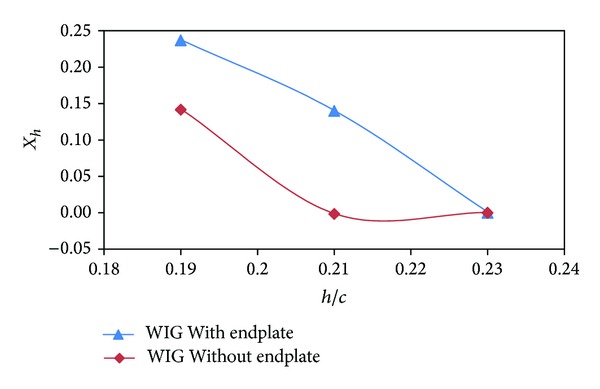
Height of aerodynamic center (*X*_*h*_) of WIG craft model with and without endplates versus ground clearance (*h*/*c*) at angle of attack of 4°.

**Figure 12:**
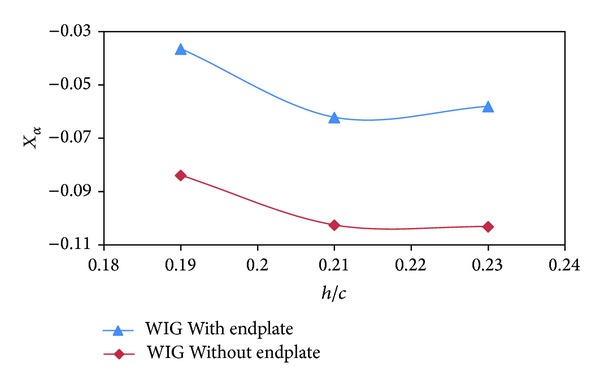
Pitch aerodynamic center (*X*_*α*_) of WIG craft model with and without endplates versus ground clearance (*h*/*c*) at angle of attack of 4°.

**Figure 13:**
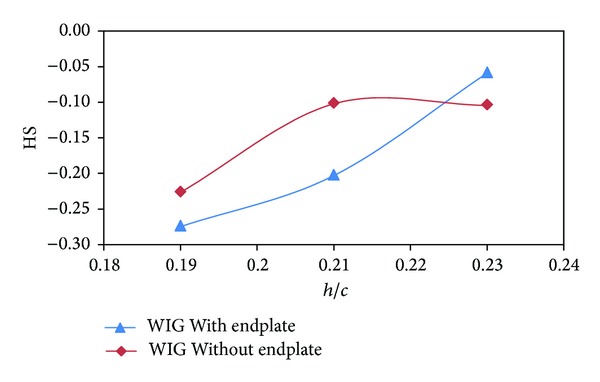
Height of static stability (HS) of WIG craft model with and without endplates versus ground clearance (*h*/*c*) at angle of attack of 4°.

As discussed earlier, static stability depends on the rate of change of moment and lift coefficient with respect to angle of attack and ground clearance. The height of aerodynamic center (*X*_*h*_) and pitch aerodynamic center (*X*_*α*_) of the WIG craft model with endplates were upstream than that of the WIG craft model without endplates as depicted in Figures 11 and 12. The height of static stability (HS) of the WIG craft model for both with and without endplates was negative with respect to ground clearance (Figure 13) that makes the craft statically stable for both cases [15].

## 6. Conclusion

This study experimentally investigated the aerodynamic characteristics of a WIG craft model with a new configuration of a compound wing. Effect of endplates on the craft aerodynamic characteristics was studied and it was found that endplates increase the lift and drag ratio of the WIG craft model. Finally, the following points can be concluded from this research work.

- A new design of WIG craft was presented by combining compound wing with new configuration and endplates.
- The aerodynamic performance of the new design was investigated by wind tunnel tests and aerodynamic coefficients are presented for the new design.
- The static stability of the new design was investigated and it was found that the new design is statically stable enough.

## Figures and Tables

**Table 1:** Principle dimensions of WIG craft.

Scale factor	1 : 6
Wing span	83.4 cm
Wing root chord	66.7 cm
Middle wing span	41.4 cm
Aspect ratio	1.25
Anhedral angle	13°
Length of WIG craft	1.2 m
Breadth of WIG craft	0.13 m
Tail wing span	0.78 m
Tail wing chord	0.15 m

## Nomenclature

*c*: Chord length
*C*_*L*_: Lift coefficient
*C*_*D*_: Drag coefficient
*C*_*M*_: Moment coefficient
*D*: Drag corce
*h*: Height of trailing edge above the ground
*h*/*c*: Ground clearance
HS: Height of static ctability
*L*: Lift force
*L*/*D*: Lift-to-drag ratio
*S*: Planform area of wing
*X*_*cp*_: Center of pressure
*U*: Freestream velocity
*α*: Angle of attack, geometric angle of incidence.
